# Genome-wide identification of UDP-glycosyltransferases in the tea plant (*Camellia sinensis*) and their biochemical and physiological functions

**DOI:** 10.3389/fpls.2023.1191625

**Published:** 2023-06-06

**Authors:** Timothy D. Hoffmann, Elisabeth Kurze, Jieren Liao, Thomas Hoffmann, Chuankui Song, Wilfried Schwab

**Affiliations:** ^1^ Biotechnology of Natural Products, Technische Universität München, Freising, Germany; ^2^ State Key Laboratory of Tea Plant Biology and Utilization, Anhui Agricultural University, Hefei, Anhui, China; ^3^ International Joint Laboratory on Tea Chemistry and Health Effects, Anhui Agricultural University, Hefei, Anhui, China

**Keywords:** tea plant, Camellia sinensis, secondary plant metabolites, UDP glycosyltransferases, glycosides

## Abstract

Tea (*Camellia sinensis*) has been an immensely important commercially grown crop for decades. This is due to the presence of essential nutrients and plant secondary metabolites that exhibit beneficial health effects. UDP-glycosyltransferases (UGTs) play an important role in the diversity of such secondary metabolites by catalysing the transfer of an activated sugar donor to acceptor molecules, and thereby creating a huge variety of glycoconjugates. Only in recent years, thanks to the sequencing of the tea plant genome, have there been increased efforts to characterise the UGTs in *C. sinensis* to gain an understanding of their physiological role and biotechnological potential. Based on the conserved plant secondary product glycosyltransferase (PSPG) motif and the catalytically active histidine in the active site, UGTs of family 1 in *C. sinensis* are identified here, and shown to cluster into 21 groups in a phylogenetic tree. Building on this, our current understanding of recently characterised *C. sinensis* UGTs (CsUGTs) is highlighted and a discussion on future perspectives made.

## The role of tea: past and present

With an over thousand-year long history that spans numerous countries, tea (*Camellia sinensis* (L.) O. Kuntze) is one of the oldest tree crop species (Meegahakumbura et al., 2018). According to legend, the origin of the tea culture began around 2737 B.C. when leaves of the tea tree fell into a pot with boiling water. China’s second emperor Shen Nung was immediately fascinated by the pleasant scent and drank the intriguing brew (Harbowy and Balentine, 1997). Tea was first cultivated 2100 years ago and the infusion with hot water of the leaves is known since the Western Han Dynasty (207 B.C. – 9 A.D.) (Lu et al., 2016). In traditional Chinese medicine the tea plant was used as a herbal medicine and as a stimulant for promoting digestion, detoxification, regulation of blood sugar and body temperature, and healing of wounds (Chopade et al., 2008).

Today it is the most popular caffeine-containing beverage across the world, and after water, the most frequently consumed drink (Schneider and Segre, 2009; Xia et al., 2017). In the year 2021 the worldwide tea production was an estimated 6.5 million tonnes (FAO, 2022). Besides China, India and Kenya (the three largest producers with an approximate 2.74, 1.26 and 0.57 million tonnes respectively in 2020), tea is currently grown in 50 countries demonstrating its immense economic importance (Ridder, 2022b). Despite of sustainability challenges, the demand for tea all over the world is growing rapidly with a rate of 5% every year (Jayasinghe and Kumar, 2019). The global tea industry reached a value of approximately 229.3 billion USD in the year 2022 (Ridder, 2022a).

The post-harvest processing is what affects the composition of the final tea leaf product as, black, green, white, oolong and Pu-erh tea are all produced from the fresh leaves of the same plant (Alcázar et al., 2007). After the harvesting of fresh leaves, the processing comprises withering, rolling, and fermentation steps, while enzymatic oxidation reactions are responsible for the characteristic aroma and colour of tea **(** Palmer, 1984 **)**. The three major classes of tea are the unfermented green, semi-fermented oolong and fully-fermented black tea (Yang et al., 2013). With a global annual production of 76 - 78%, black tea is the most consumed form worldwide. The remaining 20 - 22% are produced as green tea, which is mainly consumed in Asia and North Africa, followed by less than 2% as oolong tea (McKay and Blumberg, 2002; Alcázar et al., 2007). Due to its attractive aroma, pleasant taste, health-promoting benefits and medicinal effect, resulting from the content of secondary plant metabolites, tea has a high medicinal as well as cultural significance (Hilal and Engelhardt, 2007).

## The tea plant

*Camellia sinensis* is an evergreen perennial shrub or small tree that belongs to the genus *Camellia* within the flowering plant family Theaceae, and can grow naturally up to 15 m high (Wei et al., 2018). However, a bush height of up to 1 m is maintained for appropriate cultivation and harvesting conditions (Mondal et al., 2004). The white coloured flowers appear individually or in pairs at the axils. Approximately 5 years after planting, the plant starts bearing green fruits containing 2 to 3 seeds. Both the leaves and the leaf buds are used for the production of tea (Chaturvedula and Prakash, 2011).

Out of 119 species belonging to the genus *Camellia*, the family of Theaceae comprises further economically important species such as *C*. *japonica* and *C*. *reticulata* with their attractive flowers, as well as *C*. *oleifera*, a traditional oil tree for production of high-quality edible seed oil (Xia et al., 2017). The breeding history of the tea plant is over 1000 years old and resulted in a large number of land races and elite cultivars that were grown and selected from naturally occurring seed sources (Meegahakumbura et al., 2018). Cultivated *C*. *sinensis* plants have two distinct tea varieties: the China type tea *C*. *sinensis* var. *sinensis* and the Assam type tea *C*. *sinensis* var. *assamica* from which all kinds of tea originated from (Wachira et al., 2013). The slow-growing shrubs of *C*. *sinensis* var. *sinensis* with small leaves are able to tolerate cold climates. Therefore, this variety is adaptable to a broad geographic range, and has become the most popular elite tea tree cultivar in China. In contrast, the quick-growing tea plant of *C*. *sinensis* var. *assamica* with large leaves and high sensitivity to cold weather is primarily grown in tropical and subtropical regions such as Yunnan Province, China and India (Willson and Clifford, 2012). More than 600 cultivated tea varieties are available with distinctive properties such as disease tolerance, drought or frost resistance, or high contents of certain compounds such as caffeine (Mondal et al., 2004). Especially with respect to biotic and abiotic stress such as climate change, cultivars have to adapt to different habitat conditions to ensure tea productivity and quality in the future.

Compared to other essential crops (e.g. rice), research on functional genomics of the tea plant was lagging behind for a long time (Xia et al., 2020). Especially for modern breeding, the use of genetic resources is required. Through the successful next-generation sequencing of the tea plant genome in 2017 and 2018, significant progress in enabling the genomic and genetic study of the plant has been made (Xia et al., 2017; Wei et al., 2018).

## Glycoside precursors determine the aromatic qualities of tea leaves

In plants, the process of the biosynthesis and emission of particular low molecular-weight volatile compounds is developmentally regulated and serves different functions in the organism (Dudareva et al., 2004). While fresh leaves are odorless or show a slight smell (especially the sweet and floral tea), it is the aroma developed during the tea manufacturing process by endogenous enzymes that is a crucial factor affecting the preferences of consumers for evaluating the character and quality of tea products (Mizutani et al., 2002). In fresh tea leaves, many aromatic compounds occur in forms of water-soluble glycosides or non-volatile precursors that are finally liberated due to glycosidases during the tea processing (Zheng et al., 2016). The monoterpene alcohols geraniol and linalool as well as the aromatic alcohols 2-phenylethanol and benzyl alcohol are released from their respective glycosides and pose the predominant volatile compounds that contribute to the specific floral aroma of oolong tea and black tea (Ho et al., 2015). The grassy note present in green tea can be attributed to (*Z*)-3-hexenol (Ohgami et al., 2015). Linalool and (*Z*)-3-hexenol are on the one hand responsible for the aromatic qualities of tea leaves, on the other hand, they are involved in tea plant defence responses to herbivore attack as leaves emit various volatiles in high concentrations (Dong et al., 2011).

**Figure 1:**
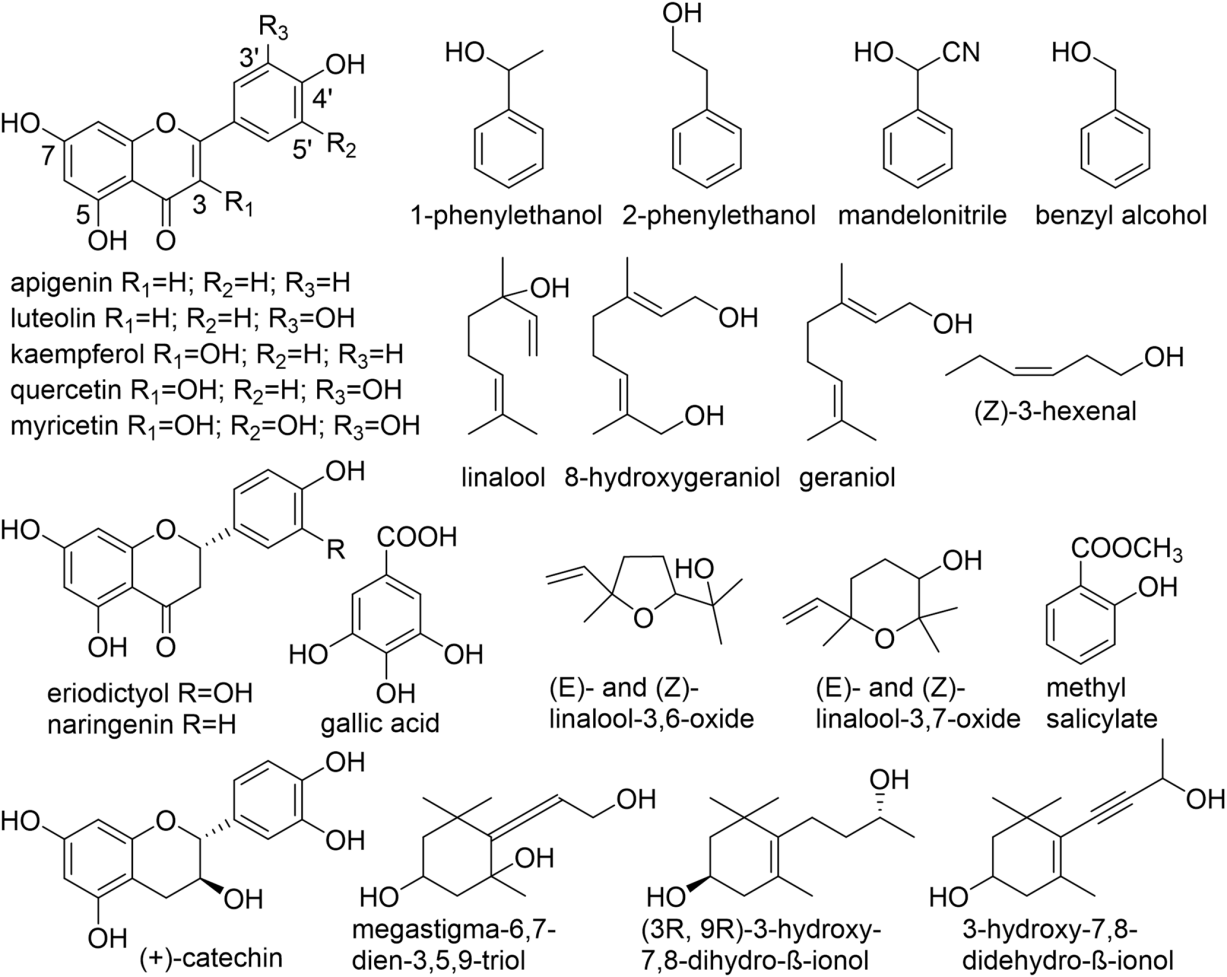
Chemical structures of aglycones found in *C*. *sinensis*. (Guo et al., 1993; Guo et al., 1994; Kobayashi et al., 1994; Moon et al., 1994; Moon et al., 1996; Nishikitani et al., 1996; Nishikitani et al., 1999; Wang et al., 2000; Ma et al., 2001; Kinoshita et al., 2010; Yang et al., 2013; Zhou et al., 2014; Plumb et al., 1999).

Numerous non-volatile, water-soluble, glycosidically bound volatiles (GBV) that accumulate in fresh tea leaves, were identified, structurally investigated and studied in detail (**Figure 1**, **Table 1**). Due to their frequently low abundance in plant tissues as well as a lack of chromophores, molecules consisting of a sugar unit linked to a small volatile compound escaped detection for a long time and thus represent a relatively new class of plant secondary products (Song et al., 2018).

**Table 1:** Selected small molecule glycosides found in *C*. *sinensis*.

Phenols		Volatiles	
Aglycone	Sugars	Aglycone	Sugars
apigenin	di-6,8-*C*-diglucose	benzyl alcohol	glucose
	6-*C*-glucose-8-*C*-arabinose		glucose-6’-*O*-xylose
	6-*C*-arabinose-8-*C*-glucose	(*Z*)-3-hexenol	glucose
	5-*O*-glucose-4’-*O*-rhamnose		glucose-6’-*O*-xylose
	8-*C*-glucose-2’-*O*-rhamnose	geraniol	glucose
	6-*C*-glucose		glucose-6’-*O*-xylose
	8-*C*-glucose		glucose-6’-*O*-arabinose
	6-*C*-glucose-2’-*O*-glucose	(*E*)- and (*Z*)-linalool 3,6-oxide	glucose
	6-*C*-glucose-7-*O*-glucose		glucose-6’-*O*-xylose
luteolin	6-*C*-glucose	(*E*)- and (*Z*)-linalool 3,7-oxide	glucose
	8-*C*-glucose		glucose-6’-*O*-xylose
myricetin	3-*O*-galactose-6-*O*’’-rhamnose		glucose-6’-*O*-apiose
	3-*O*-glucose-2-*O*’’-rhamnose	2-phenylethanol	glucose
	3-*O*-galactose		glucose-6’-*O*-xylose
	3-*O*-glucose	1-phenylethanol	glucose
	3-*O*-glucose-6-*O*’’-rhamnose		glucose-6’-*O*-xylose
quercetin	3-*O*-glucose	methyl salicylate	glucose
	3-*O*-galactose		glucose-6’-*O*-xylose
	3*-O-*galactose-*O*’’-glucose-6-*O*’’’-rhamnose	mandelonitrile	glucose
	3-*O*-glucose-*O*’’-glucose-6-*O*’’’-rhamnose	megastigma-6,7-dien-3,5,9-triol	glucose
	3-*O*-glucose-6-*O*’’-rhamnose	3-hydroxy-7,8-didehydro-β-ionol	glucose
	3-*O*-hexose-arabinose-rhamnose-glucose	8-hydroxygeraniol	glucose-6’-*O*-xylose
	3-*O*-hexose-hexose-rhamnose-glucose	(*S*)-linalool	glucose-6’-*O*-xylose
	7-*O*-rhamnose-3-*O*’’-hexose-rhamnose-glucose	(3*R*, 9*R*)-3-hydroxy-7,6-dihydro-β-ionol	glucose-6’-*O*-apiose
	3-*O*-glucose-6’’-*O*-rhamnose-3’’’-*O*-glucose		
	3-*O*-galactose-6’’-*O*-rhamnose-3’’’-glucose		
kaempferol	3-*O*-glucose		
	3-*O*-galactose		
	3-*O*-galactose-*O*’’-glucose-6-*O*’’’-rhamnose		
	3-*O*-glucose-*O*’’-glucose-6-*O*’’’-rhamnose		
	3-*O*-glucose-6-*O*’’-rhamnose		
	3-*O*-hexose-arabinose-rhamnose-glucose		
	3-*O*-hexose-hexose-rhamnose-glucose		
	7-*O*-rhamnose-3-*O*’’-hexose-rhamnose-glucose		
	3-*O*-glucose-6’’-*O*-rhamnose-3’’’-*O*-glucose		
	3-*O*-galactose-6’’-*O*-rhamnose-3’’’-*O*-glucose		
eriodictyol	di-5,3-*O*-glucose		
	7-*O*-glucose		
naringenin	di-*O*-glucose		
gallic acid	glucose		
catechin	7-*O*-rhamnose		
	3-*O*-galactose		
	3-*O*-glucose-6’’-*O*-rhamnose		

(Finger et al., 1991; Engelhardt et al., 1993; Dou et al., 2008; Dai et al., 2015; Jiang et al., 2015; Chen et al., 2018; Price et al., 1998; Plumb et al., 1999; Scharbert et al., 2004; Wu et al., 2012).

GBVs are mostly present in forms of β-D-glucosides and β-primeverosides (6-*O*-β-D-xylopyranosyl-β-D-glucopyranoside; glucose-6-*O*-xylose) (Wang et al., 2001b; Wang et al., 2001a). The hydrolysis of aroma precursors of ß-primeverosides, catalysed by a unique disaccharide-specific glycosidase present in tea plant, the ß-primeverosidase, causes the release of various aroma compounds (Mizutani et al., 2002). However, the content of aroma glycosides that exists in form of disaccharides is higher than that of monoglucosides due to the higher content of primeverosides (Wang et al., 2000). Furthermore, tea leaves also accumulate significant amount of flavonoid *O*- and *C*-glycosides (Engelhardt et al., 1993; Peterson et al., 2005).

## UDP-glycosyltransferases catalyse the formation of glycosides in the tea plant

The glycosylation of small volatile compounds is a common modification process of naturally occurring plant secondary metabolites and widespread in the plant kingdom (Wang and Hou, 2009). As a key reaction glycosylation determines the chemical complexity of natural substances and further effects the chemical stability and water solubility while simultaneously reducing the chemical reactivity and toxicity (Bowles et al., 2005). Physiologically, glycosylation facilitates inter- and intracellular transport, storage and accumulation in plant cells (Tiwari et al., 2016).

The accumulation of aroma or flavour compounds, natural colorants, phytohormones and phytoanticipins in form of glycosides occurs in various organs such as flowers, fruits, or leaves (Markham et al., 2001; Zagrobelny et al., 2004; Husar et al., 2011; Song et al., 2016).

In plants, the transfer of sugars is catalyzed by glycosyltransferases (EC 2.4.x.y). According to sequence identity, the consensus sequences, and catalytic specificity, GTs can be classified into more than 115 protein families (http://www.cazy.org/GlycosylTransferases.html) (Ross et al., 2001). Uridine diphosphate (UDP) glycosyltransferases (UGTs) catalyze the transfer of an activated nucleotide diphosphate sugar (usually UDP-glucose, UDP-D-glucuronic acid, UDP-D-xylose, UDP-L-rhamnose, and UDP-galactose) to acceptor aglycones with high stereo- and regiospecificity. Saccharides, polypeptides or proteins, lipids, nucleic acids, antibiotics or low molecular weight compounds (known as secondary metabolites) are potential acceptor molecules for UGTs (Yonekura-Sakakibara and Hanada, 2011; Dewitte et al., 2016). Numerous UGT candidates belong to the GT family 1 and glycosylate small molecules (Paquette et al., 2003). As a product, *O*-, *S*-, *N*- and *C*-glycosides as well as sugar esters are formed (Schwab et al., 2015). Besides *O*-glycosides, tea plants are a rich source of *C*-glycosides and thus C-UGTs (**Table 1**) (Sang et al., 2011; Su et al., 2018). *C*-glycosides are more stable compared to their respective aglycones and *O*-glycosides (Xiao et al., 2016). Furthermore, they exhibit a wide range of health-beneficial effects which make them attractive for medicinal chemistry (Choi et al., 2014).

**Figure 2:**
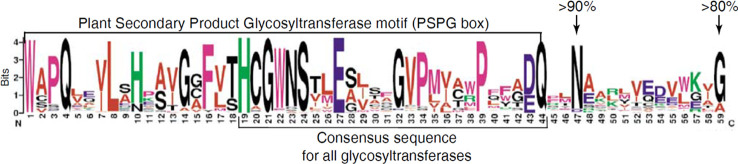
Conserved protein sequence of the Plant Secondary Product Glycosyltransferase motif (PSPG box).

Plant UGTs that are involved in glycosylation of secondary metabolites have a common signature motif of ~44 amino acids near the C-terminus called PSPG motif (Plant Secondary Product Glycosyltransferase) (**Figure 2**) (Hughes and Hughes, 1994). The amino acid residues comprising the PSPG-box have been shown to be involved in binding of the UDP moiety of the activated sugar. This motif is conserved in all UGTs of higher plants including *C*. *sinensis* (Bowles et al., 2005; Offen et al., 2006). In addition, a His residue has been identified in the N-terminus, which acts as a catalytic base together with an Asp residue (Offen et al., 2006). These features can therefore be used to screen plant genomes for UGT genes and the enzymes they encode.

UGTs have various biological roles in plants. They glycosylate phytohormones and thus are involved in plant growth and development as well as the adaptation to environmental stresses (Hou et al., 2004; Tognetti et al., 2010; Husar et al., 2011). The linkage of a sugar moiety with small molecules enhances the stability and thus prevents degradation (Chen et al., 2011; Yonekura-Sakakibara and Hanada, 2011). Furthermore transport and storage processes into the vacuole are mediated by glycosylation (Richman et al., 2004; Ostrowski and Jakubowska, 2014). *Via* glycosylation plants developed an efficient mechanism to neutralize toxic endogenous metabolites produced by plant-pathogens, pollutants, and xenobiotics (Ahn et al., 2011; Schweiger et al., 2013; Tiwari et al., 2016). UGTs acting on plant volatiles have an important role in plant defence and stress tolerance, highlighting their function in plant protection (Jing et al., 2019) as well as plant resistance to pathogens (Chong et al., 2002; Wang and Hou, 2009).

Studies over recent years have underlined the significance of the GT superfamily, however the availability of biochemical data on individual member enzymes is still limited, hindering the understanding of their function in plants. In the Protein Data Bank, more than 150 GT crystal structures are listed (https://www.rcsb.org/) but among them, there are less than 30 plant UGTs. To date, only a few combinations of volatiles and their responsible UGTs have been functionally characterized (Song et al., 2018). The presence of hundreds of genes coding for UGTs in tea plant and their interesting substrate spectrum would give new insights into the biochemistry, function and physiological role of enzyme members.

Although *C. sinensis* UGTs (CsUGT) have a relevant role in tea plant performance and determine the quality of the tea product, little is known about their physiological roles. With the constantly increasing number of plant genome sequences, as well as transcriptome data and metabolite profiles, it is possible to identify and verify genes of novel glycosyltransferases and further characterize their catalytic ability to form small-molecule glycosides (Song et al., 2018). The availability of the tea plant genome sequence (Xia et al., 2017; Wei et al., 2018) presents the possibility to generate a collection of CsUGT that would greatly enhance the scientific research on tea glycosides.

This review provides an overview of UGTs from the tea plant and introduces the biochemically characterized representatives and their biological significance.

## Functionally characterized UGTs from the tea plant

**Figure 3:**
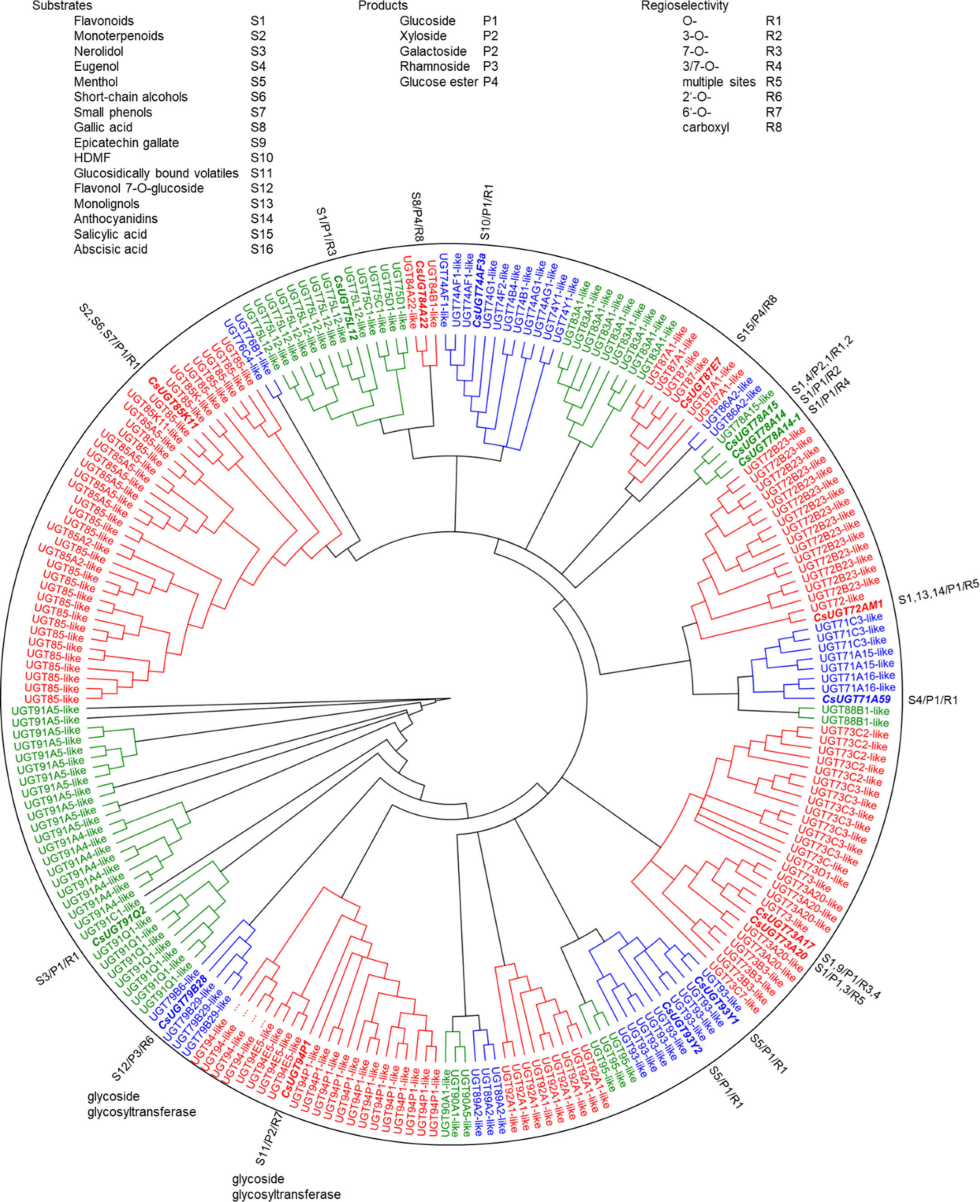
Phylogenetic analysis of 276 *C. sinensis* family 1 glycosyltransferases. Twenty-one major UGT groups were found. The neighbour joining tree was constructed by Geneious Basic 5.6.7 software with 1000 replications. Substrates, products, and regioselectivity of biochemically characterized UGTs are indicated.

Glycosyltransferase and glucosyltransferase protein sequences were retrieved from the Tea Plant Information Archive by name search (http://tpdb.shengxin.ren/), aligned and manually inspected for the presence of the PSPG motif, catalytically active His and the co-activating Asp residue. Biochemically characterized, literature-known tea UGTs, were added and the UGT80 subfamily, known to glycosylate sterols, were deleted because of their additional sequence length encoding the structure responsible for anchoring into membranes. Similarly, putative protein modifying UGTs and presumed polysaccharide-forming enzymes were removed. A phylogenetic tree was constructed from the remaining 230 sequences (**Figure 3**). The sequences were 400 to 637 amino acids long and were clustered into 15 subgroups (**Table 2**) designated A to R, with the groups N and O found to be absent in tea plant (Li et al., 2001; Caputi et al., 2012; Wilson and Tian, 2019). The R group was the most recent identified group, and all the genes in this group are sensitive to stresses (methyl jasmonate treatment, cold stress, salt stress and drought stress) and, are highly expressed in different tissues (Cui et al., 2016). At this point, it is worth noting the inconsistencies in the literature in assigning the groups to the different UGT classes. Wilson and Tian (2019) and Yang et al. (2022) assigned UGT95, which was assigned to group R here according to Cui et al. (2016), to groups Q and O, respectively.

**Table 2:** Number of UGT members in different crop plants and model plants.

Species	Phylogenetic groups																		References
	A 79, 91, 94	B 89	C 90	D 73	E 71 72 88	F 78	G 85	H 76	I 83	J 87	K 86	L 74 75 84	M 92	N	O 93	P	R 95	Total	
*Malus x domestica*	34	0	8	11	50	3	50	17	12	13	7	15	8	3	3	8	0	242	(Zhou et al., 2017)
*Camellia sinensis*	56	3	3	27	27	4	38	2	9	7	2	29	9	0	11	0	3	230	This study
*Vitis vinifera*	25	4	6	9	45	8	29	7	13	7	2	23	5	1	3	0	0	228	(Wei et al., 2021)
*Populus trichocarpa*	12	2	6	14	49	0	42	5	5	6	2	23	6	1	3	2	0	178	(Caputi et al., 2012)
*Arabidopsis thaliana*	14	3	3	13	22	3	6	19	1	2	2	17	1	1	0	0	0	107	(Li et al., 2001)

The UGTs of tea plant were retrieved from the Tea Plant Information Archive (TPIA; http://tpdb.shengxin.ren/) by name search using the terms glycosyltransferase and glucosyltransferase.

The tea plant *C. sinensis* contains a similar number of UGT genes as other woody crops such as *Malus x domestica* and *Vitis vinifera*, whereas *Populus trichocarpa* and especially the weed *Arabidopsis thaliana* have significantly fewer UGT genes (**Table 2**). The woody plants are particularly rich in group G UGT85 genes, which encode GBV-producing enzymes and others (Ohgami et al., 2015; Song et al., 2018). A distinctive feature of the tea plant is the high number of group A UGT79, UGT91, and UGT94 genes. This is reflected in the large number of distinct di- and even trisaccharide glycosides in tea leaves (**Table 1**), the formation of which, among others, is catalyzed by UGT94 enzymes (Ohgami et al., 2015). These UGTs are thus, classified as glycoside specific glycosyltransferases (GGTs), which specifically catalyse sugar-sugar glycosylation with a high regioselectivity. They contribute to the formation of di/triglycosides, and are typically composed of families UGT79, UGT91 and UGT94 (Ono et al., 2020).

The first report on the identification of a UGT from the tea plant was not published until 2014 (Ohgami et al., 2014). High-throughput RNA sequencing of fresh tea leaves, followed by rapid amplification of cDNA ends (RACE), yielded the first full-length cDNA of a UGT from the tea plant. The recombinant UGT73A17 protein catalyzed 3-*O*-glucosylation and, to a lesser extent, 7-*O*-glucosylation of the flavonol quercetin. UDP-glucose was the preferred donor substrate. The preferential expression of the *UGT73A17* gene in mature leaves and the concomitant accumulation of quercetin-3-*O*-glucoside indicated that UGT73A17 is involved in part in the biosynthesis of flavonol glucosides.

The same group also isolated the first UGTs involved in the glycosylation of volatile organic compounds (VOCs) from the tea plant (Ohgami et al., 2015). Tea plants store numerous volatile organic compounds such as benzyl alcohol, 2-phenylethanol, (*Z*)-3-hexenol, linalool, and geraniol as water-soluble disaccharide glycosides, mainly as ß-primeverosides (6-*O*-ß-D-xylopyranosyl-ß-D-glucopyranosides). These glycosides are formed by sequential glucosylation and xylosylation catalyzed by CsUGT85K11 and CsUGT94P1, respectively. CsUGT85K11 showed promiscuity for the acceptor substrate as it catalyzed the glucosylation of monoterpenes, aromatic and aliphatic alcohols, but selectivity for the donor substrate UDP-glucose. On the other hand, CsUGT94P1 preferentially xylosylated the sugar moiety of geranyl glucoside at the 6’-hydroxy group, but was also able to use UDP-glucose as a donor substrate, albeit with one-third reduction in activity, and can therefore also be classed as a GGT. *CsUGT94P1* gene expression correlated with flavonoid content in shoots of two metabolically distinct tea cultivars (Liu et al., 2023).

In 2016, 178 UGT genes were identified in a *C. sinensis* transcriptome dataset, greatly facilitating the isolation and characterization of full-length UGT genes from the tea plant (Cui et al., 2016). The number of UGT genes in this dataset was significantly lower than that of UGT genes from the genome database because not all UGT genes are expressed at a given time point. After manual inspection and removal of putative non-functional genes, 132 candidate CsUGTs remained, from which CsUGT84A22, CsUGT78A14, and CsUGT78A15 proteins were functionally active and catalyzed the formation of β-glucogallin (gallic acid glucose ester), flavonol 3-*O*-glucosides, and flavonol 3-*O*-galactoside, respectively (Cui et al., 2016). The Gln373 position proved to be crucial for 3-*O*-glucosyltransferase activity while the genes appear to be involved in the biosynthesis of astringent compounds in *C. sinensis* (Cui et al., 2016). *CsUGT78A14* and *CsUGT78A15* genes were overexpressed in the model plants *Arabidopsis thaliana* and *Nicotiana tabacum* (Jiang et al., 2018). In both transgenic plant species, *CsUGT78A14* promoted flavonol 3-*O*-glucoside production, whereas flavonol 3-*O*-galactosides accumulated in *CsUGT78A15* transgenic plants. In a multiomics analysis, CsUGT78A15 protein content correlated with flavonoid content in the shoots of two metabolically distinct tea genotypes (Liu et al., 2023).

Later, *CsUGT78A14* was found to be strongly induced by cold stress in *C. sinenesis*, and two allelic forms of the gene were isolated (Zhao et al., 2019). While the CsUGT78A14-1 protein produced mainly kaempferol-3,7-di-*O*-glucoside in addition to the corresponding monoglucosides, CsUGT78A14-2 catalyzed primarily the formation of the 3-*O*-glucoside. The amino acid sequences of the two proteins differ only by an Ala residue at position 438. The accumulation of kaempferol glycosides was consistent with *CsUGTA14* gene expression levels in response to cold stress. Down-regulation of the gene resulted in reduced tolerance of *C. sinensis* to cold stress, probably due to a reduced ability to scavenge reactive oxygen species.

The transcriptome dataset of the tea plant was also the starting point for the isolation and characterization of a flavonoid 7-*O*-glucosyltransferase (CsUGT75L12; (Dai et al., 2017)). In *Arabidopsis* plants overexpressing *CsUGT75L12*, levels of flavonol 7-*O*-glucosides were increased, while levels of quercetin, kaempferol, and flavan-3-ols were reduced (Jiang et al., 2018). The encoded protein was analyzed in detail by site-directed mutagenesis (Dai et al., 2017). Residues His56 and Thr151, corresponding to the catalytically active His and activating Asp in other UGTs, could each be replaced by Ala without compromising activity. Instead, Gln54 appears to play a key role in enzymatic activity. Also in a UGT from *Glycine max*, His15 and Asp125 were not crucial for enzyme activity (Noguchi et al., 2007). Furthermore, enzymes of the UGT84 family, such as the aforementioned CsUGT84A22, lack Asp because they catalyze the glycosylation of acids that are already present as anions, i.e. further activation of the catalytically active His is not necessary (Huang et al., 2018). Multiomics analysis of shoots showed that *CsUGT75L12* gene expression correlated with flavonoid content in two metabolically distinct tea cultivars (Liu et al., 2023).

Bitter and astringent tasting flavonoid 7-*O*-neohesperidosides are biosynthesized in tea plants through the sequential 7-*O*-glucosylation of flavonoids catalysed by CsUGT75L12 and 2’-*O*-rhamnosylation catalysed by CsUGT79B28 (Dai et al., 2022). The accumulation patterns of flavonoid 7-*O*-glucosides and 7-*O*-neo-hesperidosides correlate with the expression levels of *CsUGT75L12* and *CsUGT79B28*, respectively. CsUGT79B28 is a further example of a GGT.

CsUGT72AM1 was able to glucosylate not only flavonols but also flavanones, dihydroflavonols, anthocyanidins, and monolignols (Zhao et al., 2017a; He et al., 2018). Thus, it shows promiscuity for the acceptor substrate and forms multiple products, as 3-, 4-, 7-, and 4’-*O*-glucosides were detected. When the anthocyanidin cyanidin was used as an acceptor substrate, substrate inhibition kinetics were observed. *CsUGT72AM1* is strongly expressed in a purple-leaf tea cultivar that accumulates remarkable amounts of anthocyanins and flavonol glucosides (He et al., 2018). Because of its high specificity constant (k_cat_ K_M_^-1^) *in vitro*, CsUGT72AM1 may be involved in lignin production similar to AtUGT72E1 (Lim et al., 2005), but its substrate promiscuity suggests additional functions *in vivo*.

*CsUGT72B23* was detected in mesophyll cells of *C. sinensis* by means of a gene co-expression network based on a single-cell transcriptome atlas (Wang et al., 2022). The encoded protein transferred a glucose unit from UDP-glucose to the gallic acid residue of epicatechin gallate and epigallocatechin gallate. CsUGT73A17 also produced epicatechin gallate glucoside, but in small amounts with the binding site of the sugar not analyzed in detail (Su et al., 2018).

In addition, the *CsUGT73A20* gene was identified in the tea transcriptome database (Zhao et al., 2017b). The recombinant protein was produced and functional characterization revealed broad substrate tolerance to several flavonoids but high specificity for the donor substrate UDP-glucose. CsUGT73A20 glucosylated several hydroxyl groups of acceptor substrates and produced mainly 3- and 7-*O*-glucosides, but disaccharides were also found. The regioselectivity was pH-dependent. The results of overexpression of *CsUGT73A20* in tobacco and *Arabidopsis* plants indicated that the encoded enzyme might function as a 3- and 7-*O*-rhamnosyl- and glucosyltransferase in plants, because quercetin and kaempferol rhamnosides and glucosides accumulated in the transgenic plants (Jiang et al., 2018). At the same time, there was a drop in the level of flavan-3-ols. Flavonol glycosides have been reported to improve the ability of plants to adapt to adverse environmental conditions (Li et al., 1993).

Similarly, the CsUGT73A17 enzyme exhibited broad acceptor substrate tolerance as it glucosylated flavonols, flavones, flavanones, isoflavones, and epicatechin gallate (Su et al., 2018). The 7-*O*-glucosides were the major products. Since the expression level of CsUGT73A17 increased significantly at 50°C, the enzyme might be involved in the heat response of the tea plant.

In addition to their proposed role in plant stress response, UGTs may have other biological functions. Thus, *CsUGT74B1* was identified as a differentially expressed gene by comparative transcriptome analysis of self-pollinated and cross-pollinated pistils, suggesting a role for this enzyme in the mechanism of self-incompatibility in tea plants (Ma et al., 2018). Recently, competition between anthocyanin and kaempferol glycoside biosynthesis was shown to affect pollen tube growth and seed set in *Malus* (Chen et al., 2021).

However, tea UGTs not only glucosylate phenolics including flavonoids, in recent years a number of CsUGTs have also been functionally characterized that glycosylate plant volatiles (Jiang et al., 2018; Chen et al., 2020; Jing et al., 2020; Zhao et al., 2020a; Zhao et al., 2020b; Kurze et al., 2021; Zhao et al., 2022). Plants produce airborne signalling metabolites such as (*Z*)-3-hexenol in response to herbivore attack (Wei and Kang, 2011). Subsequently, formed hydroxylated volatiles can be converted to glycosides in the producer plant and recipient plants using UGTs (Sugimoto et al., 2014). This mechanism allows the growth and survival rate of herbivores to be suppressed even in neighbouring plants. Three allelic enzymes CsUGT85A53-1,2 and 3 from tea plant showed high activity toward (*Z*)-3-hexenol, and overexpression in tobacco significantly increased the level of the corresponding glucoside (Jing et al., 2019). In addition, airborne (*Z*)-3-hexenol upregulated the expression level of *CsUGT85A53* in the tea plant.

Expression of *CsUGT85A5*3 was also strongly induced by various abiotic stresses, and the encoded protein was found in the cytoplasm and nucleus (Jing et al., 2020). Ectopic expression of the gene in *Arabidopsis* resulted in reduced transcription levels of the flowering repressor gene FLC and an activator of FLC ABI5 in transgenic plants, leading to an early flowering phenotype. The CsUGT85A53 protein glucosylated abscisic acid (ABA) *in vitro* and *in planta*, which was confirmed by overexpression of the corresponding gene in *Arabidopsis*. Application of ABA restored the early-flowering phenotype in the *CsUGT85A53*-overexpressing lines, which had higher germination rates than the controls. Thus, *CsUGT85A53* promotes the transition to flowering as a positive regulator through an ABA-controlled mechanism.

The sesquiterpene nerolidol appears to play a role in the response of tea plants to cold stress, as the accumulation of nerolidol glucosides was induced by cold stress, consistent with the increased expression level of *CsUGT91Q2* in different tea cultivars (Zhao et al., 2020b). The encoded protein showed nerolidol glucosyltransferase activity, and downregulation of the gene in *C. sinensis* resulted in lower levels of nerolidol glucoside and compromised the cold tolerance of the tea plant. Similar to the experiments with (*Z*)-3-hexenol, the tea plants were able to take up the sesquiterpene from the air and transform it into the glucoside. Nerolidol may play a role in triggering communication between plants in response to cold stress.

Low temperature treatment also results in accumulation of eugenol glucoside in *C. sinensis*, and analysis of cold stress-induced UGT genes yielded *CsUGT78A15* whose protein was able to glucosylate the phenolic compound (Zhao et al., 2020a). When eugenol was used as an acceptor substrate, UDP-glucose was the preferred donor substrate, followed by UDP-galactose and UDP-glucuronic acid, although CsUGT78A15 had been characterized as a flavonoid 3-*O*-galactosyltransferase in a previous study (Cui et al., 2016). Down-regulation of *CsUGT78A15* resulted in lower levels of eugenol glucoside under cold stress, implying a role for the encoded enzyme in the biosynthesis of eugenol glucoside at low temperatures.

Cold and drought stress trigger the expression of *CsUGT71A59*, whose encoded protein specifically glucosylates eugenol *in vitro* and *in vivo*, resulting in the formation of eugenol glucoside (Zhao et al., 2022). Down-regulation of *CsUGT71A59* gene expression in *C. sinensis* reduced eugenol glucoside content and impaired cold and drought stress tolerance of plants. Exposure of tea plants to airborne eugenol induced *CsUGT71A59* expression, increased eugenol content, and enhanced cold tolerance by modulating the accumulation of reactive oxygen species. Drought tolerance was improved by altering abscisic acid homeostasis and stomatal closure. Eugenol and its glucoside thus play a role in tolerance to cold and drought whereby CsUGT78A15 and CsUGT71A59 can produce the glucoside.

In fruits, 4-hydroxy-2,5-dimethylfuran-3(2H)-one (HDMF) is an important odorant and contributes to the caramel-like notes of some teas. HDMF has been identified in tea plants and two allelic proteins CsUGT74AF3a and b have been characterized that catalyze the formation of HDMF glucoside (Chen et al., 2020). Site-directed mutagenesis identified an amino acid at the C-terminus Ala456Val responsible for donor substrate preference. The transcript levels of *CsUGT74AF3* correlated with the accumulation of HDMF glucoside in different tea cultivars, and down-regulation of the gene in tea leaves decreased the level of HDMF glucoside compared with the levels in controls. Enzymes of different UGT families are able to glucosylate HDMF, but a protein from the UGT74 family has not been described yet (Effenberger et al., 2019).

Although menthol, an important aroma chemical, has not previously been found as a natural constituent of the tea plant, CsUGT93Y1 and CsUGT93Y2 were identified as (+/-)-menthol glucosyltransferases in a whole-cell biotransformation screen (Kurze et al., 2021). The results demonstrate once again that several enzymes involved in the transformation of secondary metabolites exhibit substrate promiscuity.

The expression of *CsUGT87E7* was significantly induced by the application of salicylic acid (SA), a plant hormone that plays an important role in the establishment of basal resistance, and infection with the tea pathogen *Pseudopestalotiopsis camelliae-sinensis* (Hu et al., 2022). The encoded protein glucosylated SA and produced the SA glucose ester (SGE). Down-regulation of the gene in the tea plant resulted in lower levels of SGE and greater susceptibility to pathogen infection compared to control plants. *CsUGT87E7*-silenced *C. sinensis* leaves accumulated less SA after pathogen infection and showed lower expression of pathogenesis-related genes. Thus, CsUGT87E7 is an SA carboxyl-glucosyltransferase involved in plant disease resistance by modulating SA homeostasis. The CsUGTs characterised to date have been summarised in **Table 3**.

**Table 3:** Characterised Glycosyltransferases from *C. sinensis*.

Group	Enzyme	Genetic Source	Host Characterisation	Sugar Donor	Acceptor(s)	Product(s)	Reference
A	UGT79B28	*C. sinensis*	*E. coli* (*in vitro* assays) *N. benthamiana* (*in vivo* expression)	UDP-Rha (others tested)	Flavonoid 7*-O-*glucosides e.g. Naringnin-7*-O-*glucoside Apigenin-7*-O-*glucoside Quercetin-7*-O-*glucoside Luteolin-7*-O-*glucoside	Flavonoid 7*-O-*di-glycosides	(Dai et al., 2022)
	UGT91Q2	*C. sinensis*	*E. coli* (*in vitro* assays) *C. sinensis* (*in vivo* suppression)	UDP-Glc	Nerolidol (further substrates, 60 tested in total)	Nerolidol glycoside	(Zhao et al., 2020b)
	UGT94P1	*C. sinensis*	*E. coli* (*in vitro* assays)	UDP-Xyl (others tested)	Geranyl *O*-β-D-glucopyranoside (Specific activity; xylosylation of the 6’-hydroxy group of geranyl β-D-glucopyranoside, other aroma glucosides tested)	Geranyl *O*-β-primeveroside	(Ohgami et al., 2015)
D	UGT73A17	*C. sinensis* var *sinensis* cv. Yabukita	*E. coli* (*in vitro* assays)	UDP-Glc (others tested)	Quercetin (others tested)	Quercetin 3-*O*-β-D-glucopyranoside	(Ohgami et al., 2014)
	UGT73A17	*C. sinensis*	*E. coli* (*in vitro* assays)	UDP-Glc	Flavonols: kaempferol, quercetin and myricetin Flavones: apigenin, luteolin and tricetin Flavanone: naringenin Isoflavones: genistein and epicatechin gallate (17 flavonoids tested in total)	Major products 7-*O*-glucosides	(Su et al., 2018)
	UGT73A20	*C. sinensis*	*E. coli* (*in vitro* assays) *N. tabacum* (*in vivo* expression)	UDP-Glc (others tested) UDP-Rhm (*in vivo*)	Flavonoids: Kaempferol, quercetin, myricetin, naringenin, apigenin and kaempferide *In vivo*: Flavanols and flavanol monoglucosides	Flavonoid 3*-O-*glucosides/7*-O-*glucosides Kaempferol 7*-O-*glucoside (dominant at pH 8) Kaempferol 3*-O-*glucoside (dominant at pH 9) *In vivo:* Q3G7Rha, Q3Rha7Rha, K3G7Rha and K3G	(Zhao et al., 2017a)
	UGT73A20	*C. sinensis*	*A. thaliana* (*in vivo* over expression) *N. tabacum* (*in vivo* expression)	UDP-Glc	Kaempferol Quercetin	Flavonoid 3*-O-*glucosides/7*-O-*glucosides: kaempferol-3*-O-*rhamnoside (K-3-R) kaempferol-3, 7-di*-O-*rhamnoside (K-3-R-7-R) K-3-R, quercetin-3*-O-*glucoside (Q-3-G), quercetin-3*-O-*rhamnoside (Q-3-R), quercetin-3,7-di*-O-*rhamnoside, quercetin-3,7-di*-O-*rhamnoside (Q-3-R-7-R)	(Jiang et al., 2018)
E	UGT71A59	*C. sinensis*	*E. coli* (*in vitro* assays) *C. sinensis* (*in vivo* suppression)	UDP-Glc (preferred) UDP-Gal UDP-Glu	Eugenol Further acceptors: Vanillin, geraniol, 4-hydroxycoumarin, ABA, salicylic acid, pyrogallic acid, and jasmonic acid, among other	Eugenol glucoside	(Zhao et al., 2022)
	UGT72AM1	*C. sinensis* var ‘Mooma1’	*E. coli* (*in vitro* assays)	UDP-Glc	Quercetin Cyanidin (also Kaempferol and Myricetin)	Quercetin 3-*O*-glucoside Cyanidin 3-*O*-glucoside	(He et al., 2018)
	UGT72AM1	*C. sinensis*	*E. coli* (*in vitro* assays)	UDP-Glc	Flavonols: kaempferol, quercetin, myricetin Flavanones: naringenin, eriodictyol Phenolic acid: coniferyl aldehyde	Flavonoid 3-*O*-glucosides Coniferyl aldehyde 4-*O*-glucoside Naringenin 7-*O*-glucoside and 4’-*O*-glucoside	(Zhao et al., 2017a)
	UGT72B23	*C. sinensis* var. *sinensis* cv. *‘Shuchazao’*	*E. coli* (*in vitro* assays) *N. benthamiana* (*in vivo* expression) *C. sinensis* (*in vivo* suppression)	UDP-Glc	Epicatechin gallate (ECG) Epigallocatechin gallate (EGCG) Further acceptors: Myricetin, 4-Hydroxycoumnarin, catechins (GCG), quercetin dehydrate, vanillin, naringenin, pelargonidin, kaempferol	ECG-Glucoside CGCG-Glucoside	(Wang et al., 2022)
F	UGT78A14	*C. sinensis*	*E. coli* (*in vitro* assays)	UDP-Glc (preferred) UDP-Gal	Kaempferol Quercetin Myricetin	Kaempferol-3-*O*-glycoside Quercetin-3*-O-*glycoside Myricetin-3*-O-*glycoside	(Cui et al., 2016)
	UGT78A14	*C. sinensis*	*A. thaliana* (*in vivo* expression) *N. tabacum* (*in vivo* expression)	UDP-Glc	Quercetin Kaempferol	Quercetin 3*-O-*glucoside Kaempferol 3*-O-*glucoside (also accumulated: K-3-G-R-7-G, and Q-3-G-R-7-G)	(Jiang et al., 2018)
	UGT78A14-1 & UGT78A14-2	*C. sinensis* var. *sinensis* cv. Shuchazao	*E. coli* (*in vitro* assays) *C. sinensis* (*in vivo* suppression)	UDP-Glc (preferred, others tested *in vitro*)	Kaempferol Quercetin Myricetin (53 further substrates tested *in vitro*)	Flavonol 3*-O-*glucosides UGT78A14-2: Kaempferol mono glucoside UGT78A14-1: Kaempferol 3,7-di glucoside (3- and 7*-O-*glucoside)	(Zhao et al., 2019)
	UGT78A15	*C. sinensis*	*E. coli* (*in vitro* assays)	UDP-Gal (preferred) UDP-Glc	Kaempferol Quercetin Myricetin	Kaempferol-3*-O-*glycoside Quercetin-3*-O-*glycoside Myricetin-3*-O-*glycoside	(Cui et al., 2016)
	UGT78A15	*C. sinensis*	*A. thaliana* (*in vivo* over expression) *N. tabacum* (*in vivo* over expression)	UDP-Gal	Quercetin Kaempferol	Falavanol-3*-O-*glycosides (e.g. Q-3-Gal-R-7-G, K-3-Gal-R-7-G and K-3-Gal-R) (New glycosides: Q-3-Gal-7-R, K-3-Gal-7-R)	(Jiang et al., 2018)
	UGT78A15	*C. sinensis*	*E. coli* (*in vitro* assays) *C. sinensis* (*in vivo* suppression)	UDP-Glc (preferred) UDP-Gal UDP-Glu	Eugenol Quercetin Kaempferol (52 acceptor substrates tested total)	Flavonoid-3*-O-*glucosides Eugenol glucoside	(Zhao et al., 2020a)
G	UGT85A53	*C. sinensis*	*E. coli* (*in vitro* assays) *A. thaliana* (*in vivo* expression)	UDP-Glc	ABA	ABA-glucoside	(Jing et al., 2020)
	UGT85A53-1 &, UGT85A53-2 & UGT85A53-3	*C. sinensis*	*E. coli* (*in vitro* assays) *N. benthamiana* (*in vivo* expression)	UDP-Glc (preferred) UDP-Gal UDP-Glu	(*Z*)‐3‐hexenol Further acceptors: (*E*)-3-hexanol, 1-hexanol, benzyl alcohol, 2-phenylethanol, geraniol, nerol, linalool oxide and citronellol, among other	(Z)‐3‐hexenyl glucoside	(Jing et al., 2019)
	UGT85K11	*C. sinensis*	*E. coli* (*in vitro* assays)	UDP-Glc (others tested)	Geraniol (Broad activity towards monoterpene, aromatic, and aliphatic alcohols)	Geranyl *O*-β-D-glucopyranoside	(Ohgami et al., 2015)
	UGT85K11	*C. sinensis*	*E. coli* (*in vitro* and *in vivo* assays)	UDP-Glc	(±)-menthol	(±)-menthyl-*O*-β-D-glucopyranoside	(Kurze et al., 2021)
J	UGT87E7	*C. sinensis*	*E. coli* (*in vitro* assays) *C. sinensis* (*in vivo* suppression)	UDP-Glc (others tested)	Salicylic acid	Salicylic acid glycoside Salicylic acid glucose ester	(Hu et al., 2022)
L	UGT74AF3a & UGT74AF3b	*C. sinensis*	*E. coli* (*in vitro* assays) *N. benthamiana* (*in vivo* expression) *C. sinensis* (*in vivo* suppression)	UDP-Glc (preferred) UDP-Gal UDP-Glu	HDMF EHMF HMF (further substrates tested)	HDMF-glucoside EHMF-glucoside HMF-glucoside	(Chen et al., 2020)
	UGT75L12	*C. sinensis*	*E. coli* (*in vitro* assays) *A. thaliana* (*in vivo* expression)	UDP-Glc UDP-Gal	Flavonoids: naringenin, apigenin, flavanol and genistein Flavonoid mono-glucosides: Kaempferol 3*-O-*glucoside and Quercetin 3*-O-*glucoside	*in vitro*: Flavonoid 7*-O-*glycosides *in vivo:* Quercetin 3*-O-*rhamnoside-7*-O-*glucoside and Kaempferol 3*-O-*rhamnoside-7*-O-*glucoside	(Dai et al., 2017)
	UGT75L12	*C. sinensis*	*A. thaliana* (*in vivo* over expression) *N. tabacum* (*in vivo* over expression)	UDP-Glc	Quercetin Kaempferol	Quercetin-3*-O-*rhamnoside-7*-O-*glucoside and Kaempferol-3*-O-*rhamnoside-7*-O-*glucoside	(Jiang et al., 2018)
	UGT75L12	*C. sinensis*	*E. coli* (*in vitro* assays) *N. benthamiana* (*in vivo* expression)	UDP-Glc (preferred) UDP-Gal	Flavonoids: Naringenin, Apigenin, Luteolin, Kaempferol and Quercetin Flavonoid mono-glucosides: Kaempferol 3*-O-*glucoside and Quercetin 3*-O-*glucoside	Flavonoid 7*-O-*glucosides	(Dai et al., 2022)
	UGT84A22	*C. sinensis*	*E. coli* *(*in vitro* assays)	UDP-Glc (preferred) UDP-Gal	Gallic acid Benzoic acids: p- hydroxybenzoic acid, benzoic acid, and syringic acid Cinnamic acids: cinnamic acid, p-coumaric acid, caffeic acid, ferulic acid, and sinapic acid	Galloyl-β-D-glucose ester Syringoyl-β-D-glucose ester Cinnamoyl-β-D-glucose ester p-Coumaroyl-β-D-glucose ester Caffeoyl-β-D-glucose ester Feruloyl-β-D-glucose ester Sinapoyl-β-D-glucose ester	(Cui et al., 2016)
O	UGT93Y1	*C. sinensis*	*E. coli* (*in vitro* and *in vivo* assays)	UDP-Glc	(±)-menthol (+)-Isomenthol Fenchyl alcohol (+)-Neomenthol	(±)-menthyl-β-D-glucopyranoside	(Kurze et al., 2021)
	UGT93Y2	*C. sinensis*	*E. coli* (*in vitro* and *in vivo* assays)	UDP-Glc	(±)-menthol	(±)-menthyl-β-D-glucopyranoside	(Kurze et al., 2021)

Characterised glycosyltransferases form *C. sinensis*. UDP-Glc; UDP-glucose, UDP-Gal; UDP-galactose, UDP-Glu; UDP-glucuronic acid, UDP-Xyl; UDP-xylose, UDP-Rha; UDP-rhamnose. The table lists a selection of donors, acceptors, substrates and products of each respective CsUGT and, for more detailed information references are provided.

## UGT expression analysis

**Figure 4:**
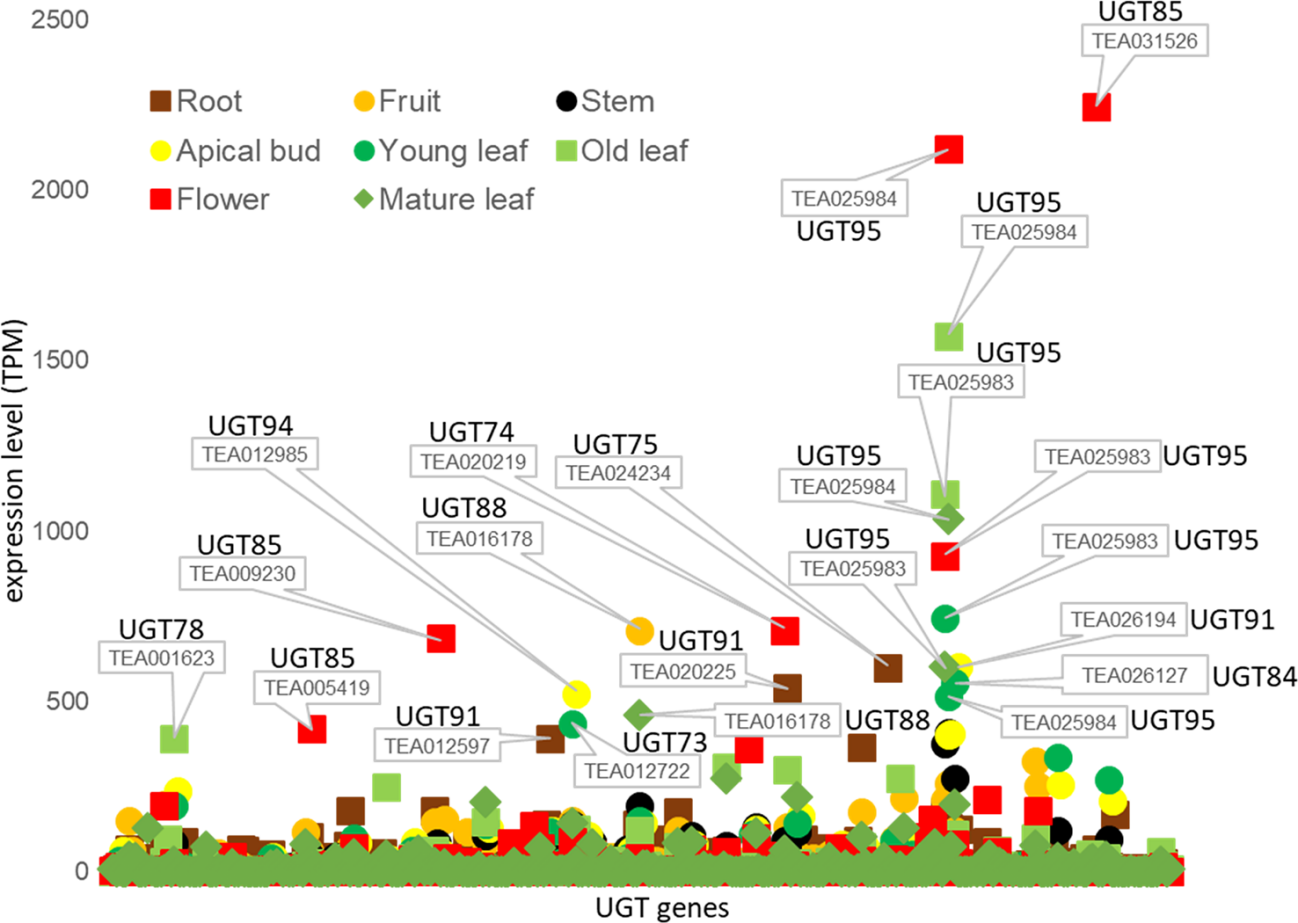
Tissue specific expression of UGT genes of *C. sinensis* family 1 glycosyltransferases. Expression levels (TPM) of UGT genes in eight tissues of the tea plant was extracted from the Tea Plant Information Archive (TPIA, http://tpdb.shengxin.ren/). RNA-seq data was acquired from eight representative tissues of tea plant, including apical buds, young leaves, mature leaves, old leaves, immature stems, flowers, young fruits and tender roots (Wei et al., 2018).

We also studied transcriptome data from the Tea Plant Information Archive (TPIA, http://tpdb.shengxin.ren/) and examined the expression levels of the various UGT genes in eight different tissues of *C. sinensis* (**Figure 4**). None of the UGT genes of the enzymes already biochemically characterized were among the 20 most highly expressed transcripts. Two of the three UGT95 genes of the tea genome (TEA025983 and TEA025984) showed the highest expression levels. These are tea-specific genes that are rarely found in other plant species. The encoded proteins catalyze the *O*-glycosylation of flavonoids (Wilson and Tian, 2019). None of the three UGT95 genes from *C. sinensis* has been previously studied. Abundant mRNA levels of one of the two UGT88 and one of the three UGT84 genes were also found. The products of these genes can form *O*-, *N*-, *S*-glycosides, and sugar esters (Wilson and Tian, 2019). The encoded protein of *CsUGT84A22*, a paralog of TEA026127, generates β-glucogallin, the glucose ester of gallic acid (Cui et al., 2016). Furthermore, several UGT85 and UGT91 genes were among the most highly expressed. These produce glucosides of volatile metabolites and form flavonoid glycosides (Wilson and Tian, 2019). Particularly high transcript levels of individual UGT genes were quantified in flowers (TEA031526, 025984, and 025983) and old leaves (TEA025984 and 025983), while mRNA levels in stems were relatively low.

**Figure 5:**
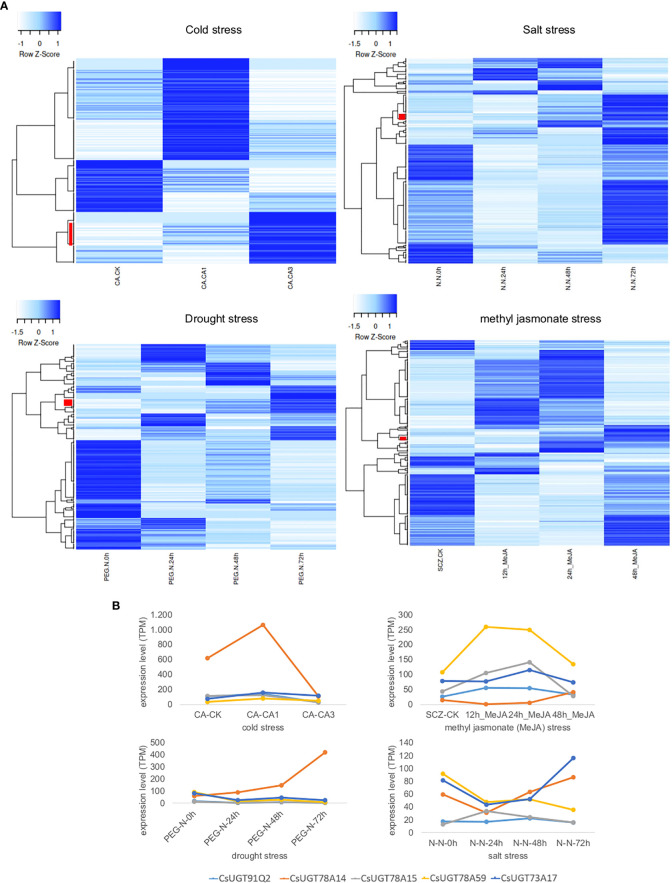
Transcriptome heatmap of family 1 UGTs from *C. sinensis* under different abiotic stress conditions and plant hormone treatment. Data was retrieved from the Tea Plant Information Archive (TPIA, http://tpdb.shengxin.ren/). **(A)** Cold tolerance: RNA-Seq reads were collected from leaves of tea plant at three stages of cold acclimation (CA) process, including nonacclimated (CK), fully acclimated (CA1) and de-acclimated (CA3) (Wang et al., 2013). Salinity stress: RNA-seq reads were collected from leaves of tea plant under salt stress (Zhang et al., 2017). A 200 mM NaCl solution was used to simulate salt-stress conditions for *C. sinensis* plant within 0, 24, 48 and 72 h. Drought tolerance: RNA-seq reads were collected from young leaves of tea plant subjected to four stages of drought stress: 25% polyethylene glycol (PEG) treatment for 0, 24, 48 and 72 h (Zhang et al., 2017). Methyl jasmonate (MeJA) treatment: RNA-seq data from tea plant leaves in response to MeJA treatment were adopted from (Shi et al., 2015). Leaves were treated with MeJA for 0, 12, 24, and 48 hours. Red bars indicate *UGT* genes whose expression levels correlate directly with treatment duration. **(B)** Detailed expression levels of CsUGT genes whose gene products have already been biochemically characterized and shown to be involved in stress responses; in light blue *CsUGT91Q2*, in orange *CsUGT78A14*, in grey *CsUGT78A15*, in yellow *CsUGT78A59*, and in dark blue *CsUGT73A17*.

Furthermore, UGT expression in tea plants under different stress conditions (cold, salt, and drought) and plant hormone treatment (methyl jasmonate) was investigated by RNA-Seq analysis. The data was taken from the Tea Plant Information Archive (TPIA, http://tpdb.shengxin.ren/). The different treatments resulted in different changes in UGT gene expression, yielding stress-specific patterns (**Figure 5**). Under cold stress, the expression of 26 UGT genes correlated directly with treatment duration, while under salt, drought, and methyl jasmonate treatments, this was only 8, 11, and 6 genes, respectively. Cold stress strongly increased the transcript levels of several UGT74 and UGT85 genes in particular as a function of stress duration, but also those of representatives of the UGT73, UGT79, UGT83, and UGT91 classes. CsUGT91Q2 has previously been shown to modulate cold stress tolerance in *C. sinensis* (Zhao et al., 2020b). Furthermore, *CsUGT78A14*, *CsUGT78A15*, and *CsUGT71A59* have also been characterized as cold stress-induced genes whose gene products can improve the cold tolerance of tea plants (Zhao et al., 2019; Zhao et al., 2020a; Zhao et al., 2022). Two UGT74 and UGT72 genes each were up-regulated in addition to one UGT71 and UGT83 gene each as a function of salt stress duration, while drought stress mainly up-regulated UGT72, UGT87, as well as UGT71, UGT73, UGT75, and UGT91 genes, and after methyl jasmonate treatment UGT94, UGT72, UGT73, and UGT87 genes were more strongly expressed. Consistent with these results, *CsUGT73A17* and *CsUGT71A59* have already been identified as heat- and drought-responsive genes whose gene products catalyse the formation of flavonoid and eugenol glucosides, respectively (Su et al., 2018; Zhao et al., 2022).

## Conclusion

Of the 276 family 1 UGTs of the tea plant postulated in this work, i.e. enzymes that glycosylate small molecules, only about 18 members have been functionally biochemically characterized so far. The total number of UGTs is a conservative estimate since in this work, the catalytically active His served as selection criterion but for CsUGT75L12 it could be shown that an alternative amino acid can take over this function (Dai et al., 2017). The biochemical studies showed that in addition to flavonoids, small volatile compounds such as monoterpenes, short-chain alcohols and phenols, and plant hormones are decorated with sugars by the UGTs (Ohgami et al., 2014; Ohgami et al., 2015; Jing et al., 2019; Jing et al., 2020; Zhao et al., 2020a; Hu et al., 2022). Furthermore, representatives producing disaccharide derivatives were identified (Ohgami et al., 2014; Dai et al., 2022). In general, flavonoid UGTs in particular exhibited promiscuity toward their acceptor substrates (Su et al., 2018) but showed specificity with respect to the donor substrate (Cui et al., 2016). The UGTs of tea plants, as well as other plant species, are expressed in a developmental and tissue-specific manner, with the expression further influenced by various biotic and abiotic factors (Jing et al., 2019; Zhao et al., 2020a; Zhao et al., 2020b). Glycosylation of airborne volatiles has been found to not only play a role in signalling but, in certain cases, also provide a substrate for the synthesis of defence compounds. Research on volatile reception in tomato species in response to herbivory attack in neighbouring plants has shown that a glycosyltransferase UGT91R1 is involved in glycosylation of (*Z*)-3-hexanyl β-*D*-glucopyranoside to (*Z*)-3-hexenyl β-*D*-vicianoside which consequently hampers growth of the cutworm larvae when ingested (Sugimoto et al., 2023). (*Z*)-3-Hexanol is a common component of green-leaf volatiles and is glucosylated by CsUGT85A53 (Jing et al., 2019) forming the substrate of UGT91R1, so it is feasible that the mechanism presented is more widespread in plants and could possibly be found in the tea plant. Glycosylation therefore seems to play a larger and more general role in the priming of plant defences and as such proves an important target for future research. Recent studies on tea UGTs showed that the expression of UGTs induced by various stressors, such as drought and cold, and the resulting formation of glycosides can improve the resistance of plants to these environmental stresses (Zhao et al., 2019; Zhao et al., 2022). These mechanisms are directly or indirectly influenced by plant hormones but are poorly understood (Jing et al., 2020; Hu et al., 2022). In addition, the glucosylated metabolites could themselves function as signalling agents. A more detailed understanding on which glycosyltransferases are involved and how they specifically contribute to plant stress resistance will provide genetic targets that could be modulated to improve plant fitness. This could occur, for example, through the production of toxic chemicals that deter pests, or through faster priming of other plant defences towards imminent abiotic or biotic stress factors. However, further research is needed to be able to better adapt tea plants to the environmental changes that will take place in the coming years.

## Future perspective: glycosides with modifications - the case of sulphates

Glycosides can undergo modifications such as malonylation, further glycosylation, and even the addition of sulphate groups by sulfotransferases (Hashiguchi et al., 2014; Song et al., 2018). While malonylated glycosides haven not yet been identified in the tea plant, to our knowledge, sulphated glycosides have. In *C. sinensis* the sulphated glycosides Isovitexin 2”-sulfate (Prechafuroside A) and Vitexin 2”-sulfate (Prechafuroside B) are thought to be the precursors to the flavone *C*-glycosides Chafurosides A and B (**Table 4**) (Ishida et al., 2009). Chafuroside A and B are unusual in that they contain a condensed dihydrofuran ring. This is thought to form when Prechafuroside is exposed to heat and, *via* a S_N_2 mechanism and transition state, causes the deprotonation of *O*-7 which attacks C-2” causing desulfonation and subsequent cyclization. Chafuroside is found in trace amounts in Oolong tea that has been heat treated to >140°C; a post-harvesting processing step done to enhance flavour, taste, and stability of the tea leaves (Ishida et al., 2009; Kurahayashi et al., 2020). Chafurosides show potential as anti-inflammatory therapeutics and therefore, further investigation into their *in vivo* synthesis is warranted (Furuta et al., 2004; Onoue et al., 2012). While there are methods for the synthesis of Chafuroside and Prechafuroside no biological pathway has been identified for the formation of Prechafuroside (Furuta et al., 2004; Nakatsuka et al., 2004; Furuta et al., 2009; Ishida et al., 2009; Kurahayashi et al., 2020). Prechafuroside A and B are unusual in that the sulphate is bound to the glycosylated sugar and not to the flavonoid skeleton. The only similar structure to have been identified from plants is that of tricin 7*-O-*β-glucopyranoside-2”-sulphate sodium salt from *Livistona australis* (Kassem et al., 2012) (**Table 4**). In contrast sulphated flavonoids and sulphated glycosides have been identified in various plants but with the sulphate group bound to the flavonoid skeleton (Teles et al., 2018). Likely, an uncharacterised *C*-glycosyltransferase and sulfotransferase in *C. sinensis* play a role in the production of the flavone *C*-glycoside Chafuroside. Although UGT708 enzymes are involved in the production of *C*-glucosides in many other plants (Putkaradze et al., 2021; Putkaradze et al., 2023), members of other UGT classes are probably responsible for their formation in the tea plant, because UGT708 genes have not been identified in the tea plant genome (**Figure 3**). Flavonoid *C*-glycosyltransferase, such as those of the flavone apigenin, have already been identified in other plants as well as sulfotransferases that catalyse the addition of a sulphate to position 7 of the flavonoid skeleton (Hashiguchi et al., 2014; Sasaki et al., 2015; He et al., 2019; Mashima et al., 2019).

**Table 4:** Structures of glycosides with sulphated sugar moieties and their derivatives.

Name	Plant	Structure	Reference
Tricin 7*-O-*β-glucopyranoside-2”-sulphate sodium salt	*Livistona australis*	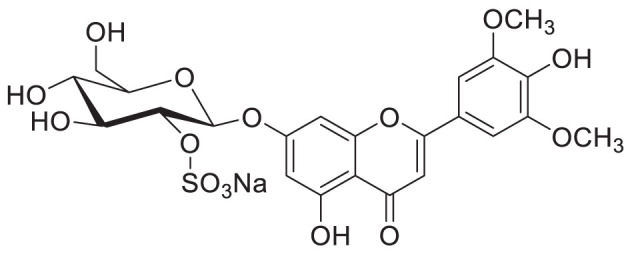	(Kassem et al., 2012)
Isovitexin 2”-sulfate	*Camellia sinensis*	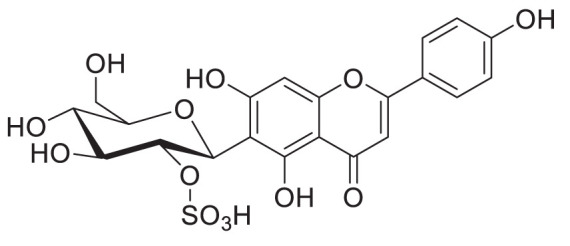	(Ishida et al., 2009)
Vitexin 2”-sulfate	*Camellia sinensis*	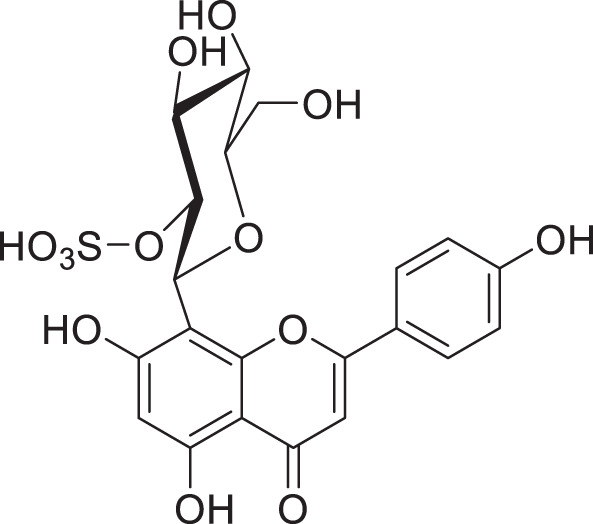	(Ishida et al., 2009)
Chafuroside A	*Camellia sinensis*	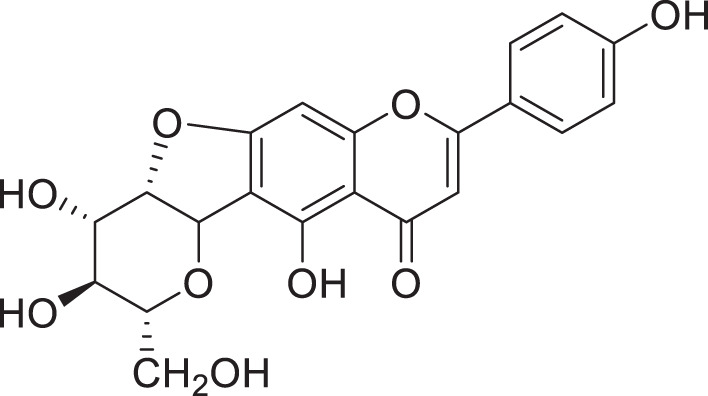	(Ishida et al., 2009)
Chafuroside B	*Camellia sinensis*	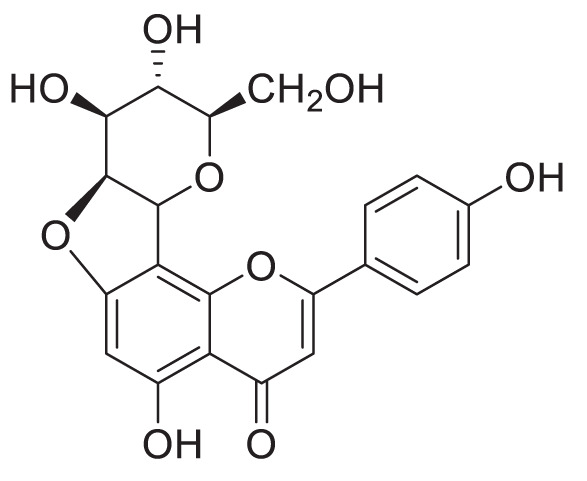	(Ishida et al., 2009)

For example the *Arabidopsis thaliana* sulfotransferase AtSULT202B7 prefers flavonoid glycosides over their aglycone counterparts, introducing a sulphate at position 7 on the flavonoid skeleton (Hashiguchi et al., 2014). While not yet reported in literature it can be reasonably assumed that glycosyltransferases with catalytic specificity towards sulphated aglycones occur in nature. Furthemore, of the few UGTs from *C. sinensis* that have been biochemically characterised (**Table 3**), all catalyse the formation of *O*-linked glycosides. Based on the presence of *C*-linked glycosides in tea (**Tables 1**, **4**) *C*-glycosyltransferases are likely to be identified in tea plants. Taken together there is a large scope for the further characterisation of CsUGTs which promise to reveal enzymes with strong biotechnological potential and, reveal candidate genes for the development of more robust plants towards the climate and environmental challenges of the next decade.

## Author contributions

TDH: Writing - Review & Editing, Conceptualization, Writing – Original Draft, Visualization. EK: Writing - Review & Editing, Conceptualization, Writing – Original Draft. JL: Writing – Review & Editing. TH: Writing – Review & Editing. CS: Writing – Review & Editing. WS: Writing - Review & Editing, Conceptualization, Writing – Original Draft, Visualization, Supervision, Funding Acquisition. All authors contributed to the article and approved the submitted version.
